# Proteomics analysis of *Trichoplusia ni* midgut epithelial cell brush border membrane vesicles

**DOI:** 10.1111/1744-7917.12547

**Published:** 2017-12-18

**Authors:** Muhammad Afzal Javed, Cathy Coutu, David A. Theilmann, Martin A. Erlandson, Dwayne D. Hegedus

**Affiliations:** ^1^ Saskatoon Research and Development Centre Agriculture and Agri‐Food Canada Saskatoon Saskatchewan Canada; ^2^ Summerland Research and Development Centre Agriculture and Agri‐Food Canada Summerland British Columbia Canada; ^3^ Department of Food & Bio‐Product Sciences University of Saskatchewan Saskatoon Saskatchewan Canada

**Keywords:** brush border membrane vesicles, midgut, proteomic analysis; *Trichoplusia ni*

## Abstract

The insect midgut epithelium is composed of columnar, goblet, and regenerative cells. Columnar epithelial cells are the most abundant and have membrane protrusions that form the brush border membrane (BBM) on their apical side. These increase surface area available for the transport of nutrients, but also provide opportunities for interaction with xenobiotics such as pathogens, toxins and host plant allelochemicals. Recent improvements in proteomic and bioinformatics tools provided an opportunity to determine the proteome of the *T. ni* BBM in unprecedented detail. This study reports the identification of proteins from BBM vesicles (BBMVs) using single dimension polyacrylamide gel electrophoresis coupled with multi‐dimensional protein identification technology. More than 3000 proteins were associated with the BBMV, of which 697 were predicted to possess either a signal peptide, at least one transmembrane domain or a GPI‐anchor signal. Of these, bioinformatics analysis and manual curation predicted that 185 may be associated with the BBMV or epithelial cell plasma membrane. These are discussed with respect to their predicted functions, namely digestion, nutrient uptake, cell signaling, development, cell–cell interactions, and other functions. We believe this to be the most detailed proteomic analysis of the lepidopteran midgut epithelium membrane to date, which will provide information to better understand the biochemical, physiological and pathological processes taking place in the larval midgut.

## Introduction

*Trichoplusia ni* (Lepidoptera: Noctuidae), also known as the cabbage looper moth, is a major agricultural pest in most of the world. *T. ni* will feed on an exceptionally broad range of plants, including over 160 species in 36 families (Sutherland & Green, 1984). Many of these plants are important crop species, including cruciferous vegetables. As a result, *T. ni* is a common and devastating pest in vegetable growing areas and greenhouses worldwide.

In lepidopteran larvae, the gut is composed of three sections: the foregut, midgut and hindgut. The foregut is lined with a chitin‐containing cuticle and facilitates physical processing of food. Posterior and anterior sphincters separate the midgut from the foregut and hindgut, respectively. The midgut is lined by the peritrophic matrix (PM), an acellular fibrous sheet composed mainly of chitin, mucopolysaccharides and proteins. The PM separates the food bolus from the midgut epithelial cell layer and prevents access by microorganisms and larger food particles to epithelial cells (Lehane & Billingsley, 1996; Hegedus *et al*., 2009). In *T. ni*, the columnar cells are the most common in the midgut epithelium. Underlying the columnar cells are regenerative cells which replenish sloughed columnar cells. Columnar cells have membranous protrusions on the apical surface called the brush border membrane (BBM) which increase the surface area of the epithelium for food absorption. Goblet cells are interspersed among the columnar cells along the midgut epithelium (Braun & Keddie, 1997).

The gut is also the primary site for many physiological, biochemical and biological interactions between the insect and the environment. The main route of entry into the host for most insect pathogens is via the gut. Insect viruses, such as the well‐studied *Autographa californica* multiple nucleopolyhedrovirus (AcMNPV), cross the PM to infect midgut columnar and regenerative cells (Granados & Lawler, 1981; Keddie *et al*., 1989; Knebel‐Morsdorf *et al*., 1996; Javed *et al*., 2016). Entry of virions into host midgut cells involves binding to cell surface receptors by virus ligands followed by fusion with the BBM (Granados & Lawler, 1981; Horton & Burand, 1993). The viral ligands are believed to be the *per os* infectivity factors; however, their cognate epithelial cell receptors, responsible for the initial interaction, have yet to be discovered (Peng *et al*., 2013). The delta‐endotoxin released by *Bacillus thuringiensis* was first shown to interact with an aminopeptidase‐N that is anchored to the epithelial cell membrane by glycosylphosphatidylinositol (GPI) (Knight *et al*., 1994; Garczynski & Adang, 1995). Subsequent toxin‐binding guided proteomic analyses revealed additional targets, which included alkaline phosphatase, cadherin, actin and V‐ATP synthase (McNall & Adang, 2003; Krishnamoorthy *et al*., 2007). The significance of the midgut epithelium extends beyond host–pathogen interactions, for example, insecticidal lectins released as part of the plant defense system interact with specific cell surface glycoproteins (Lagarda‐Diaz *et al*., 2016).

Despite the importance of the midgut epithelial cell membrane in such interactions, reports on its composition are scarce. Twenty proteins were identified from *Manduca sexta* larval midgut microvilli (Pauchet *et al*., 2009), while 74 and 86 proteins were found in BBMVs from *Aedes aegypti* (Popova‐Bulter & Dean, 2009) and *Chilo suppressalis* (Ma *et al*., 2012), respectively. It should be noted that many of the proteins identified in these studies are unlikely to be associated with the outer plasma membrane and are most likely remnants of the cytosolic and endomembrane systems. Recent improvements in protein identification technology from complex protein mixtures and the availability of large genomic and transcriptomic sequence databases provide unparalleled opportunities to determine the identities of the complete cellular and subcellular organelle protein composition. In this report, we present a detailed identification of *T. ni* BBMV proteins and the most complete set of BBMV proteins reported to date. This will enhance the understanding of interactions between the midgut epithelium and micro‐organisms, host plants, and non‐biological materials.

## Materials and methods

### Preparation of T. ni BBMVs

*T. ni* larvae were reared at 27 °C on artificial diet (per L: 128 g Pinto beans, 37 g yeast, 32 g wheat germ, 16 g alfalfa meal, 16 g ascorbic acid, 12 g agar, 11 g Wesson's salts, 4 g corn oil, 3.2 g Tween 80, 1.3 g flax oil, 0.9 g methyl 4‐hydroxybenzoate, 0.8 g sorbic acid, 0.5 g chloramphenicol, 0.5 g tetracycline, and 0.25 g alpha‐tocopherol) based on Diet 7 in Vail *et al*. (1973). BBMVs were prepared using a modification of the protocols of Wolferberger *et al*. (1987) and Abdul‐Rauf and Ellar (1999). Actively feeding 4th‐instar larvae (*n* = 10) were chilled on ice for 15 min, dissected and the PM removed from longitudinally sectioned midguts. The midguts were rinsed three times in buffer A (300 mmol/L mannitol, 5 mmol/L ethylene glycol bis(*α*‐aminoethyl ether)‐N,N‐tetracetic acid (EGTA), 17 mmol/L Tris‐(hydroxymethyl) aminomethane‐HCl [pH 7.5]) and collected in preweighed 2.0 mL tubes containing 400 μL buffer A supplemented with protease inhibitor cocktail (Roche, Laval, Quebec, Canada). Collection tubes were pre‐cooled on ice and after gut collection each tube was weighed again to determine the net weight of midgut tissue. If necessary, the midgut tissue was stored at –80 °C prior to processing. Frozen gut tissue (0.4 g) was thawed on ice and mixed with buffer A to a total volume of 2 mL and homogenized by 30 strokes of a Dounce homogenizer. An equal volume of 24 mmol/L MgCl_2_ was added to the homogenized tissue and the mixture was again homogenized (five strokes) and incubated on ice for 20 min. The mixture was centrifuged at 2500 × *g* for 15 min at 4 °C to separate midgut tissue from the BBMVs. The supernatant containing the BBMVs was removed and saved on ice and the pellet resuspended in 1 mL buffer A supplemented with protease inhibitor cocktail and the process repeated. Finally, supernatants were pooled and centrifuged at 30 000 × *g* for 30 min at 4 °C. The supernatant was discarded and the pellet containing the BBMVs was resuspended in 100 μL half‐strength ice‐cold buffer A and either used immediately or stored at –80 °C.

### Transmission electron microscopy of BBMVs

The BBMVs were immobilized on formvar‐carbon coated copper grids (200 mesh) treated with poly L‐lysine by floating the grids on a drop of BBMVs for 5 min. Excess BBMV suspension was removed by touching the edge of the grid with blotting paper and the grids were negatively stained by floating on a drop of 1% phosphotungstic acid for 30 s. Excess acid was removed with blotting paper and the grids were air‐dried at room temperature overnight. A transmission electron microscope Philips CM10 (Philips Electron Optics, Eindhoven, the Netherlands) was used to observe the BBMVs. Images were captured on a plate film camera, films developed, and scanned.

### Proteomics analysis of BBMVs

The concentration of protein in the BBMV preparation and the cell debris fraction was determined using a BCA protein assay kit (Thermo Scientific, Rockford, IL, USA). BBMVs were solubilized in protein loading buffer (2% SDS, 10% glycerol, 0.01% bromophenol blue in 60 mmol/L Tris‐HCl buffer, pH 6.8), proteins separated by electrophoresis on 12% SDS‐PAGE gels and protein bands visualized by staining with Coomassie Blue. A lane of BBMV‐enriched proteins was cut into 27 slices with each slice having about one to two prominent protein bands.

The samples were subjected to LS‐MS/MS analysis at the Genome BC Proteomics Centre, University of Victoria, Canada, as per the following procedure. Trypsin digests were performed as previously described (Parker *et al*., 2005). Briefly, gel slices were manually cut into 1 mm cubes and transferred to a Genomics Solutions Progest (DigiLab Inc., Holliston, MA, USA) perforated digestion tray. The gel pieces were destained (50/45/5 v/v methanol/water/acetic acid) prior to reduction (10 mmol/L dithiothreitol, MilliporeSigma, Oakville, Ontario, Canada) and alkylation (100 mmol/L iodoacetamide, Sigma). Modified sequencing‐grade porcine trypsin solution (20 ng/μL, Promega, Madison, WI, USA) was added to the gel slices at an enzyme/protein ratio of 1 : 50. Proteins were then digested for 5 h at 37 °C prior to collection of the tryptic digests and acid extraction of the gel slices (50/40/10 v/v acetonitrile/water/formic acid). The samples were then lyophilised and stored at –80 °C prior to analysis.

The peptide digests were separated by online reverse phase chromatography using a Thermo Scientific EASY‐nLC II system with a reverse‐phase Magic C‐18AQ precolumn (100 μm I.D., 2 cm length, 5 μm, 100Å, Michrom BioResources Inc, Auburn, CA, USA) and a reverse phase nanoanalytical column Magic C‐18AQ (75 μm I.D., 15 cm length, 5 μm, 100 Å, Michrom BioResources Inc, Auburn, CA, USA) both prepared in‐house, at a flow rate of 300 nL/min. The chromatography system was coupled online with an LTQ Orbitrap Velos mass spectrometer equipped with a Nanospray II source (Thermo Fisher Scientific, Bremen, Germany). Solvents were A: 2% acetonitrile, 0.1% formic acid; B: 90% acetonitrile, 0.1% formic acid. After a 249 bar (∼10 μL) precolumn equilibration and 249 bar (∼6 μL) nanocolumn equilibration, samples were separated by a 55 min gradient (0 min: 5% B; 45 min: 45% B; 2 min: 80% B; hold 8 min: 80% B).

The LTQ Orbitrap Fusion (Thermo Fisher Scientific) parameters were as follows: Nanoelectrospray ion source with spray voltage 2.1 kV, capillary temperature 225 °C. Survey MS1 scan *m/z* range 400–2000 profile mode, resolution 60 000 FWHM@400 *m*/*z* with AGC target 1E6, and one microscan with maximum inject time of 500 ms. Lock mass Siloxane 445.120024 for internal calibration with preview mode for FTMS master scans: on, injection waveforms: on, monoisotopic precursor selection: on; rejection of charge state: 1. The samples were analyzed by the following methods: (1) top 15 FTMS/IT‐CID method with the 15 most intense ions charge state 2–4 exceeding 5000 counts were selected for CID ion trap MSMS fragmentation (ITMS scans 2–16) with detection in centroid mode. Dynamic exclusion settings were: repeat count: 2; repeat duration: 15 s; exclusion list size: 500; exclusion duration: 60 s with a 10 ppm mass window. The CID activation isolation window was: 2 Da; AGC target: 1E4; maximum inject time: 100 ms; activation time: 10 ms; activation Q: 0.250; and normalized collision energy 35%. Common human keratin and porcine trypsin peptide masses were excluded from MS/MS selection during the analysis.

### Data analysis

A MASCOT database was generated by supplementing published transcriptome data with two in‐house libraries. The transcriptome developed by Chen *et al*. (2014) from *T. ni* cell line Tnms42 undergoing infection with AcMNPV (NCBI Accession No. PRJNA260558) contributed 70 322 unigenes. In‐house 454‐sequencing libraries from brain (NCBI Accession No. SRX1745247) and midgut (NCBI Accession No. SRX1745246) contributed 141 198 and 146 091 reads, respectively. The sequences from the three databases were combined and assembled *de novo* using the CLC Genomics Workbench (7.5.1) using a word size of 64 bp resulting in 18 972 contigs (assemblies with two or more sequences). Singletons were added by mapping the published transcriptome to the assembly, collecting the unmapped reads and adding them to the assembly contig sequences to bring the transcriptome to 58 200 sequences. These were translated in all 6 reading frames and filtered for sequences over 50 amino acids resulting in a database of 261 307 hypothetical protein sequences. Common contaminant sequences were downloaded from Maxquant.org and included in the *T. ni* MASCOT database. MS/MS analysis was performed on each slice and output as an .mgf file. All .mgf files were concatenated into a single file and the mass spectra transformed into data files which were used to search the *T. ni* MASCOT database. Protein identities were assigned using the Mascot MS/MS Ion search algorithm. Mascot search parameters were as follows: peptide tolerance: 8 ppm, fixed modification: carbamidomethyl, variable modification: oxidation, MS/MS tolerance: 0.6 Da, peptide charge 2+ and 3+, and allowed missed cleavages: 1.

BBMV proteins were annotated using the CLC Genomics Workbench plugin for Blast2GO (refseq May 19, 2015), examined for the presence of signal peptides (SignalP http://www.cbs.dtu.dk/services/SignalP/) if a start codon was present, transmembrane domains (TM) (TMHMMOL/L http://www.cbs.dtu.dk/services/TMHMMOL/L), a glycophosphatidylinositol (GPI) anchor signal (BIG‐PI: http://mendel.imp.ac.at/gpi/gpi_server.html), as well as cellular localization (TargetP: http://www.cbs.dtu.dk/services/TargetP/). Additional structural features were determined using PredictProtein (Yachdav *et al*., 2014) and HMMOL/LER (Finn *et al*., 2015).

## Results

**Figure 1:**
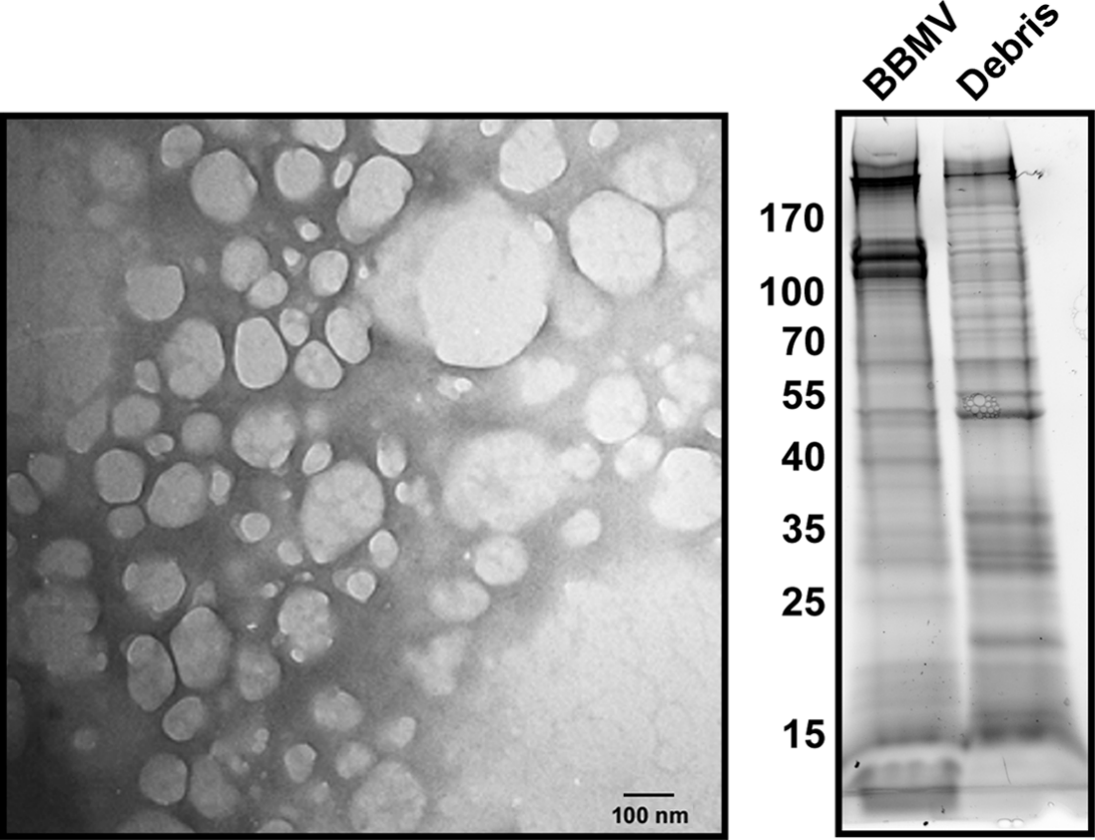
Preparation and analysis of *T. ni* midgut brush border membrane vesicles (BBMV). (A) Transmission electron microscopic image of negatively stained BBMVs. (B) SDS‐PAGE gel of proteins from 4th‐instar BBMV or cell debris remaining after BBMV isolation. The gel shows enrichment of proteins in the BBMV fraction as reflected by different protein banding patterns in the BBMV and remnant midgut cell debris preparations. The size (kDa) of molecular weight markers is shown in the left hand margin.

BBMVs were prepared from midgut tissue isolated from actively feeding 4th instar *T. ni* larvae. Separation of the *T. ni* midgut BBMV proteins by SDS‐PAGE yielded a pattern of discreet bands ranging from more than 170 kDa to less than 15 kDa in size (Fig. 1). The pattern of separated proteins in the BBMV fraction was highly distinct from that of the midgut cell debris remaining after BBMV preparation indicating that BBMV purification had been successful.

The *T. ni* BBMVs were observed by transmission electron microscopy after negative staining with phosphotungstic acid. The vesicles were circular to oval in shape and of varying sizes (ca. 50–200 nm) (Fig. 1). The morphology of the BBMVs was consistent with the morphology of BBMVs isolated from the midgut of *Pieris brassicae*, cabbage butterfly (Wolfersberger *et al*., 1987) and mosquito, *Aedes aegypti* (Abdul‐Rauf & Ellar, 1999) larvae.

### Identification BBMV proteins

A complete genome sequence is not available for *T. ni*, therefore, virtual protein sequences inferred from contigs compiled from EST sequences from all available database were used. As such, these contigs do not represent sequences from a specific strain/isolate, but rather a composite view. Genebank does not issue accession numbers for virtual genes/proteins used in these types of proteomics studies; however, all of the ESTs which contributed to building the contigs have been deposited in GenBank (see Materials and Methods) with the nucleotide sequences of the contigs and the derived protein sequences provided in Table S1 and Supporting information 1–4.

Proteomic analysis identified 3169 proteins in the BBMV fraction (Table S1; Supporting information 1 and 2). The BBMVs will contain some remnants of the cytosolic components since the process by which they are generated involves disruption of the cell membrane by homogenization followed by spontaneous formation of microvesicles that can recapture such material. To establish which proteins were likely to be associated with the BBMV plasma membrane several criteria were applied. Proteins may either be incorporated within (single and multiple pass transmembrane proteins) or attached to (GPI anchor) the outer plasma membrane. As well, outer membrane proteins often, though not exclusively, begin the trek to this destination in the endoplasmic reticulum which requires a signal peptide for entry. In total, 697 proteins were predicted to possess a signal peptide (333 proteins for which the amino terminus could be inferred from the translated EST contigs), at least one transmembrane domain (484), a GPI‐anchor signal (25), or some combination of these (Table S2; Supporting information 3 and 4). Of these, a few proteins did not show homology to any characterized proteins and, therefore, may represent newly discovered midgut proteins requiring further exploration (Table 1). Of the proteins that could be annotated, many were known or predicted to be localized to subcellular organelles (e.g., mitochondria) and were excluded from the list of putative BBMV plasma membrane proteins, while other proteins were orthologs of known plasma membrane proteins characterized in other insects and/or animals and were included. Manual curation resulted in a final set of 185 putative plasma membrane proteins that were separated into functional categories (Table 1) and are described in more detail below.

**Table 1:** Proteins associated with the *T. ni* brush border or epithelial cell membrane

Contig name	Protein description†	Signal peptide‡	Transmembrane domains	GPI signal
**1. Enzymes**
1.1 Protein metabolism
T_ni_cDNA_15821	Membrane alanyl aminopeptidase	Y	0	Y
T_ni_cDNA_15865	Membrane alanyl aminopeptidase	ND	1	Y
T_ni_cDNA_15923	Membrane alanyl aminopeptidase	ND	1	N
T_ni_cDNA_15756	Membrane alanyl aminopeptidase	ND	0	Y
T_ni_cDNA_15581	Aminopeptidase N	Y	1	Y
T_ni_cDNA_15581	Aminopeptidase N	Y	1	Y
T_ni_cDNA_15841	Zinc carboxypeptidase	ND	0	Y
T_ni_cDNA_16326	Zinc carboxypeptidase	ND	1	N
T_ni_cDNA_11989	Serine carboxypeptidase	N	1	N
T_ni_cDNA_9354	Dipeptidyl peptidase 4	ND	1	N
T_ni_cDNA_2674	Dipeptidyl peptidase 4	N	1	N
T_ni_cDNA_11394	Trypsin‐like protease	ND	1	N
T_ni_cDNA_16839	Alkaline c‐like	ND	1	N
T_ni_cDNA_15309	Alkaline c‐like	Y	1	N
T_ni_cDNA_14829	Gamma‐glutamyltranspeptidase 1	N	1	N
T_ni_cDNA_11454	Angiotensin‐converting enzyme‐like	ND	2	N
1.2 Lipid metabolism
T_ni_cDNA_15964	Bile salt‐activated lipase	ND	1	N
T_ni_cDNA_15786	Pancreatic triacylglycerol lipase	ND	1	N
T_ni_cDNA_6516	Group XV phospholipase A2	Y	1	N
1.3 Alkaline phosphatases
T_ni_cDNA_16197	Membrane‐bound alkaline phosphatase	Y	0	Y
T_ni_cDNA_7523	Membrane‐bound alkaline phosphatase	ND	0	Y
T_ni_cDNA_10461	Alkaline phosphatase	Y	1	N
1.4 Esterases
T_ni_cDNA_7581	FE4 esterase	ND	0	Y
T_ni_cDNA_5769	FE4 esterase	ND	1	N
**2. Transporters**
2.1 Nutrient transporters
T_ni_cDNA_4704	Facilitated trehalose transporter TreT1	N	12	N
T_ni_cDNA_16594	Facilitated trehalose transporter TreT1	ND	5	N
T_ni_cDNA_16422	Facilitated trehalose transporter TreT1	ND	4	N
T_ni_cDNA_16750	Facilitated trehalose transporter TreT1	N	4	N
T_ni_cDNA_6038	Facilitated trehalose transporter TreT1	ND	7	N
T_ni_cDNA_2642	Facilitated trehalose transporter TreT1	ND	6	N
T_ni_cDNA_8414	Facilitated trehalose transporter TreT1	ND	4	N
T_ni_cDNA_18685	Facilitated trehalose transporter TreT1	ND	4	N
T_ni_cDNA_5339	Facilitated trehalose transporter TreT1	N	12	N
T_ni_cDNA_10089	Facilitated trehalose transporter TreT1	Y	12	N
T_ni_cDNA_6537	Facilitated trehalose transporter TreT1	N	12	N
T_ni_cDNA_1150	Facilitated trehalose transporter TreT1‐2	ND	12	N
T_ni_cDNA_15922	Facilitated trehalose transporter TreT1‐2	N	12	N
T_ni_cDNA_15135	Facilitated trehalose transporter TreT1‐2	N	12	N
T_ni_cDNA_2589	Facilitated trehalose transporter TreT1‐2	N	12	N
T_ni_cDNA_16456	Monocarboxylate transporter 9	ND	7	N
T_ni_cDNA_15428	Monocarboxylate transporter 12 isoform x1	ND	12	N
T_ni_cDNA_7342	Monocarboxylate transporter 12	ND	5	N
T_ni_cDNA_7344	Monocarboxylate transporter 12	ND	5	N
T_ni_cDNA_936	Monocarboxylate transporter 13	N	12	N
T_ni_cDNA_15711	Peptide transporter family 1	ND	10	N
T_ni_cDNA_331	Sodium‐dependent amino acid transporter 1	ND	10	N
T_ni_cDNA_15560	Sodium‐dependent amino acid transporter 1	N	11	N
T_ni_cDNA_268	Proton‐coupled amino acid transporter 4	N	10	N
T_ni_cDNA_10643	b(+)‐Type amino acid transporter 1	ND	12	N
T_ni_cDNA_11849	Neutral and basic amino acid transport protein RBAT	N	1	N
T_ni_cDNA_484	p Protein (tyrosine transport – melanin biosynthesis)	N	4	N
T_ni_cDNA_16680	Carnitine transporter (solute carrier family 22 member 5)	ND	6	N
T_ni_cDNA_15116	Long‐chain fatty acid transport protein 4	ND	4	N
T_ni_cDNA_2856	Long‐chain fatty acid transport protein 4	N	2	N
T_ni_cDNA_16312	Folate transporter (solute carrier family 46 member 3)	ND	7	N
T_ni_cDNA_13650	Folate transporter (solute carrier family 46 member 3)	N	4	N
T_ni_cDNA_2107	Folate transporter (solute carrier family 46 member 3)	N	11	N
T_ni_cDNA_13629	Folate transporter (solute carrier family 46 member 3)	ND	5	N
T_ni_cDNA_14535	Thiamine transporter 1	N	9	N
T_ni_cDNA_6134	Sodium‐dependent multivitamin transporter	N	13	N
T_ni_cDNA_14772	Glucose transporter type 1	N	10	N
T_ni_cDNA_16283	sodium‐ and chloride‐dependent glycine transporter 1	ND	10	N
T_ni_cDNA_4340	Equilibrative nucleoside transporter 1	N	10	N
T_ni_cDNA_11410	Nucleoside transporter (solute carrier family 28 member 3)	N	6	N
2.2 Ion transporters
T_ni_cDNA_7971	Monoamine transporter (solute carrier family 22 member 3)	N	10	N
T_ni_cDNA_10297	Monoamine transporter (solute carrier family 22 member 3)	N	9	N
T_ni_cDNA_16742	Organic cation transporter (unknown specificity)	N	7	N
T_ni_cDNA_16932	Organic cation transporter (unknown specificity)	ND	3	N
T_ni_cDNA_15514	Organic cation transporter (unknown specificity)	ND	8	N
T_ni_cDNA_16584	Organic cation transporter (unknown specificity)	ND	1	N
T_ni_cDNA_16504	Organic anion transporter (solute carrier family 22 member 6)	N	12	N
T_ni_cDNA_4393	Zinc transporter zip1	N	8	N
T_ni_cDNA_15008	Zinc transporter zip1	N	7	N
T_ni_cDNA_15544	Zinc transporter 1	N	5	N
T_ni_cDNA_15075	Zinc transporter 2	ND	2	N
T_ni_cDNA_5548	Zinc transporter 5	N	6	N
T_ni_cDNA_4830	Zinc transporter 8	N	6	N
T_ni_cDNA_8634	Sodium potassium‐transporting ATPase subunit alpha	N	8	N
T_ni_cDNA_5157	Sodium potassium‐transporting ATPase subunit beta‐1	N	1	N
T_ni_cDNA_15599	Sodium potassium calcium exchanger 4	Y	11	N
T_ni_cDNA_16457	Otopetrin‐2‐like (calcium)	ND	2	N
T_ni_cDNA_8680	Plasma membrane calcium‐transporting ATPase 2	N	10	N
T_ni_cDNA_5242	Membrane magnesium transporter 1	N	2	N
T_ni_cDNA_4851	Metal transporter CNNM2	ND	4	N
T_ni_cDNA_2047	Metal transporter CNNM4	Y	5	N
T_ni_cDNA_16108	High‐affinity copper uptake protein 1	N	1	N
T_ni_cDNA_2666	High‐affinity copper uptake protein 1	N	2	N
T_ni_cDNA_1242	Copper‐transporting ATPase 1	ND	8	N
T_ni_cDNA_5956	Sodium‐independent sulfate anion transporter	ND	2	N
T_ni_cDNA_2292	Sodium‐independent sulfate anion transporter	N	9	N
T_ni_cDNA_17245	Inorganic phosphate cotransporter	N	1	N
T_ni_cDNA_9286	Inorganic phosphate cotransporter	ND	2	N
T_ni_cDNA_16781	Inorganic phosphate cotransporter	ND	5	N
T_ni_cDNA_16392	Inorganic phosphate cotransporter	ND	5	N
T_ni_cDNA_11064	Inorganic phosphate cotransporter	ND	4	N
T_ni_cDNA_11851	Inorganic phosphate cotransporter	N	10	N
T_ni_cDNA_14148	Inorganic phosphate cotransporter	N	10	N
T_ni_cDNA_8012	Band 3 anion transport protein	ND	3	N
T_ni_cDNA_8012	Band 3 anion transport protein	ND	7	N
T_ni_cDNA_11471	Band 3 anion transport protein	ND	1	N
T_ni_cDNA_8298	Bestrophin‐4 (chloride transport)	ND	5	N
T_ni_cDNA_49	Transmembrane channel‐like protein 5	N	9	N
T_ni_cDNA_6160	Transmembrane channel‐like protein 7	ND	2	N
T_ni_cDNA_5307	Transmembrane channel‐like protein 7	ND	1	N
T_ni_cDNA_3081	v‐Type proton ATPase subunit s1	Y	2	N
T_ni_cDNA_2854	v‐Type proton ATPase 16 kDa proteolipid subunit	N	4	N
T_ni_cDNA_16138	v‐Type proton ATPase 116 kDa subunit a	N	6	N
T_ni_cDNA_8655	v‐Type proton ATPase 116 kDa subunit a	N	7	N
2.3 ATP‐binding cassette (ABC) and major facilitator (MFS) superfamily transporters				
T_ni_cDNA_16946	ABC sub‐family A member 3	ND	1	N
T_ni_cDNA_6541	ABC sub‐family G member 4	ND	1	N
T_ni_cDNA_7803	ABC sub‐family G member 4	N	6	N
T_ni_cDNA_12415	ABC sub‐family G member 4	N	7	N
T_ni_cDNA_6541	ABC sub‐family G member 4	ND	5	N
T_ni_cDNA_9811	Multidrug resistance protein 1a	ND	2	N
T_ni_cDNA_7617	Multidrug resistance‐associated protein lethal 03659	N	11	N
T_ni_cDNA_16958	Multidrug resistance‐associated protein lethal 03659	ND	2	N
T_ni_cDNA_7620	Multidrug resistance‐associated protein 4	N	6	N
T_ni_cDNA_1434	Multidrug resistance‐associated protein lethal 03659	N	6	N
T_ni_cDNA_9811	Multidrug resistance protein homolog 49 isoform x1	ND	4	N
T_ni_cDNA_17333	Multidrug resistance protein homolog 49	ND	6	N
T_ni_cDNA_14948	Multidrug resistance‐associated protein 7	ND	9	N
T_ni_cDNA_3976	Multidrug resistance‐associated protein 4	ND	4	N
T_ni_cDNA_7619	Multidrug resistance‐associated protein 4	ND	5	N
T_ni_cDNA_3976	Multidrug resistance‐associated protein 4	ND	5	N
T_ni_cDNA_16436	Multidrug resistance‐associated protein lethal 03659	ND	6	N
T_ni_cDNA_11576	Multidrug resistance‐associated protein lethal 03659	N	2	N
T_ni_cDNA_3465	Major facilitator superfamily domain‐containing protein 6	N	10	N
2.4 Other transporters
T_ni_cDNA_16176	Nose resistant to fluoxetine protein 6	ND	5	N
T_ni_cDNA_16598	Nose resistant to fluoxetine protein 6	ND	3	N
T_ni_cDNA_16620	Nose resistant to fluoxetine protein 6	ND	4	N
T_ni_cDNA_16176	Nose resistant to fluoxetine protein 6	ND	4	N
T_ni_cDNA_16467	Nose resistant to fluoxetine protein 6	ND	6	N
T_ni_cDNA_11792	sid1 Transmembrane family member 1	Y	7	N
T_ni_cDNA_1279	sid1 Transmembrane family member 1	ND	4	N
T_ni_cDNA_13757	sid‐1‐Related gene3 precursor	ND	3	N
T_ni_cDNA_2198	Aquaporin	N	6	N
T_ni_cDNA_16275	Aquaporin	N	5	N
T_ni_cDNA_6828	Cell cycle control protein 50a (phospholipid transport—flippase)	ND	1	N
T_ni_cDNA_6917	Ileal sodium bile acid cotransporter‐like	Y	9	N
**3. Signaling and development**				
T_ni_cDNA_4126	G‐protein coupled receptor 125	ND	5	N
T_ni_cDNA_5794	G‐protein coupled receptor mth2‐like	ND	7	N
T_ni_cDNA_10188	Notch protein isoform X1	ND	1	N
T_ni_cDNA_8224	Dispatched protein (hedgehog signaling)	N	12	N
T_ni_cDNA_5965	Patched domain‐containing protein 3‐like (hedgehog receptor)	ND	9	N
T_ni_cDNA_15629	Fasciclin‐2 isoform x1 (adhesion protein—inhibitor of EGFR signaling)	ND	0	Y
T_ni_cDNA_15628	Fasciclin‐2 isoform x3	Y	2	N
T_ni_cDNA_10096	Fasciclin‐3 isoform x3	Y	1	N
T_ni_cDNA_8856	Plexin‐a4	ND	2	N
T_ni_cDNA_6561	Plexin domain‐containing protein 2‐like	N	1	N
T_ni_cDNA_16287	Protein rolling stone‐like	N	6	N
**4. Cell–cell interaction**				
T_ni_cDNA_1567	Integrin beta‐nu (midgut cell development)	ND	1	N
T_ni_cDNA_1572	Integrin beta‐nu	ND	1	N
T_ni_cDNA_6146	Integrin alpha‐ps1 isoform x1	ND	1	N
T_ni_cDNA_889	Integrin alpha‐ps2‐like	Y	1	N
T_ni_cDNA_1079	Dystroglycan isoform x1	ND	1	N
T_ni_cDNA_16036	Cadherin‐like membrane protein precursor	ND	1	N
T_ni_cDNA_15320	De‐cadherin‐like isoform x2	ND	1	N
T_ni_cDNA_13185	Lachesin‐like isoform x1	ND	1	N
T_ni_cDNA_1960	Innexin inx2 (gap junctions)	N	4	N
3.3 Other			1	N
T_ni_cDNA_5683	23 kDa integral membrane protein	N	4	N
T_ni_cDNA_15216	23 kDa integral membrane protein	N	4	N
T_ni_cDNA_5730	Protein mesh isoform x2	ND	1	N
T_ni_cDNA_6939	Basigin	ND	2	N
**5. Other proteins**				
5.1 Immunity/defense				
T_ni_cDNA_3905	Peptidoglycan‐recognition protein sc2‐like	N	1	N
T_ni_cDNA_3908	Peptidoglycan‐recognition protein 2‐like	N	1	N
T_ni_cDNA_14656	Croquemort‐like isoform x3	N	2	N
T_ni_cDNA_11150	Transmembrane protein 120	ND	1	N
T_ni_cDNA_6486	Transmembrane protein 120	ND	5	N
5.2 Chitin metabolism				
T_ni_cDNA_3621	Trehalase‐like isoform x1	Y	1	N
T_ni_cDNA_8547	Chitinase‐3‐like protein 2	N	1	N
T_ni_cDNA_13856	Laccase‐1‐like isoform x2	ND	0	Y
5.3 Other				
T_ni_cDNA_9349	Protein yellow	Y	0	Y
T_ni_cDNA_2522	Protein yellow‐like	ND	0	Y
T_ni_cDNA_1605	Transmembrane protein 87a	Y	7	N
T_ni_cDNA_8393	Transmembrane protein 177	ND	2	N
T_ni_cDNA_5627	Transmembrane protein 222	N	2	N
T_ni_cDNA_7343	Transmembrane protein 256 homolog isoform x1	ND	3	N
T_ni_cDNA_11297	Leucine‐rich transmembrane protein (aael003720)	ND	1	N
T_ni_cDNA_9865	Neutral ceramidase isoform x1	N	1	N
T_ni_cDNA_12371	Leucine‐rich repeat neuronal protein 3‐like isoform x1	Y	1	N
T_ni_cDNA_8987	Platelet glycoprotein v	Y	1	N
T_ni_cDNA_15370	Motile sperm domain‐containing protein 2‐like	N	2	N
T_ni_cDNA_8049	Cleft lip and palate transmembrane protein 1 homolog	N	4	N
T_ni_cDNA_13005	Cklf‐like marvel transmembrane domain‐containing protein 4	N	4	N
T_ni_cDNA_3867	Sel1 (signal transduction) or Skt5p (chitin synthesis)	ND	1	N
T_ni_cDNA_5900	TM2‐domain (related to 7 transmembrane GPCR)	ND	2	N
T_ni_cDNA_14902	Vacuolar ATPase assembly integral membrane VMA21	ND	2	N
T_ni_cDNA_6626	Uncharacterized insect protein (two protein‐binding domains)	ND	1	N
T_ni_cDNA_7146	Uncharacterized insect protein (two protein‐binding domains)	N	2	N
T_ni_cDNA_11766	Uncharacterized insect protein (NLS)	ND	1	N
T_ni_cDNA_16269	Uncharacterized insect protein	ND	0	Y

^†^Annotation based on Gene Ontology and BLAST analysis.

^‡^ND: could not be determined as 5′ open‐reading frame of the contig did not extend to the start codon; N: No; Y: Yes.

### Membrane‐associated enzymes

The initial stages of food digestion take place in the endoperitrophic space within the lumen of the lepidopteran midgut as a bolus surrounded by the PM. Here, endoproteases cleave proteins into smaller peptides that can pass through the PM into the ectoperitrophic space adjacent to the midgut epithelium. The peptides are then further processed into component amino acids by membrane‐associated amino‐ and carboxypeptidases so they may be taken up by the midgut cells. Membrane aminopeptidase N (APN) and alanyl peptidases have been isolated from lepidopteran midgut tissue (Wang *et al*., 2005) and are associated with the brush border membrane of lepidopteran midgut epithelial cells (Yuan *et al*., 2011). Four isozymes of aminopeptidase N (APN), APN1, APN2, APN3, and APN4, have been identified in *T. ni* from cDNA sequences (Wang *et al*., 2005). In this study, six BBMV plasma membrane proteins were identified that were similar to aminopeptidases classified as M1 family zinc metallopeptidases; these included two enzymes classified as APN‐like peptidases and four as alanyl aminopeptidases, although these terms are often used synonymously. In addition, two zinc carboxypeptidase proteins, a venom‐like serine carboxypeptidase and two dipeptidyl peptidases, were identified which would also contribute to the final stages of protein digestion. While endopeptidase activity is generally associated with the initial stages of digestion in the insect gut lumen, an unusual trypsin‐like protease and two alkaline C‐like endopeptidases with single transmembrane domains were identified in the BBMVs which may digest short peptides passing through the peritrophic membrane into the ectoperitrophic space (Bolognesi *et al*., 2008).

Membrane‐associated enzymes/proteins have been implicated as targets/ligands for insect pathogens or their toxins. Members of the APN family facilitate infection of the lepidopteran midgut by serving as receptors for bacterial toxins, in particular *Bacillus thuringiensis* delta‐endotoxins (Knight *et al*., 1994; Sangadala *et al*., 1994). Insect viruses also attach to, as yet, unidentified ligands on the midgut epithelium (Haas‐Stapleton *et al*., 2004); a phenomenon that precedes fusion with the epithelial membrane and that may impart host specificity (Horton & Burand, 1993). Interestingly, an alanyl APN was identified as the receptor for an insect‐borne plant virus (Linz *et al*., 2015) suggesting a role in its transmission. A proteomic study in *Plodia interpunctella* revealed that resistance to *B. thuringiensis* Cry1Ab toxin may have arisen from a decrease in the level of chymotrypsin‐like proteases required for its activation in the midgut (Candas *et al*., 2003); however, enzymes of this type were not predicted to be associated with the *T. ni* BBMV membrane in this study. Another proteomic study which examined *T. ni* cells selected in the presence of Cry1Ac toxin identified at least 30 differentially expressed enzymes/proteins; however, most of these were cytosolic or associated with cellular organelles (Gai *et al*., 2013). Comparison of BBMV proteins from resistant and susceptible insects may uncover additional *B. thuringiensis* toxin‐binding proteins.

Historically, insect lipases have been annotated according to their most closely related mammalian counterparts. Three enzymes involved in lipid digestion, including a bile salt‐activated lipase, a pancreatic triacylglycerol lipase and a group XV phospholipase A2, were associated with the BBMVs. Interestingly, the *T. ni* pancreatic lipase associated with the BBMV does not possess the cysteine‐rich C‐terminus domain, which is common to insect midgut pancreatic lipases that are associated with the PM where they may initiate digestion of dietary lipids (Simpson *et al*., 2007; Campbell *et al*, 2008; Toprak *et al*., 2015). One of the enzymes was classified as a bile salt‐activated lipase; however, insects do not produce bile salts (De Veau & Schultz, 1992). Rather, phospholipid surfactants, such as lysolecithin, may be generated by the hydrolysis of phospholipids by phospholipase A (Terra *et al*., 1994), which was also associated with the BBMVs.

Membrane‐bound alkaline phosphatases localized in the midgut of lepidopteran larvae have been well‐characterized and are used as larval midgut BBMV markers (Wolferberger *et al*., 1987; Abdul‐Rauf & Ellar, 1999). Similar to APN, midgut alkaline phosphatases are linked to the epithelial cell plasma membrane by a GPI anchor. The oligomeric form of *B. thuringiensis* delta‐endotoxin has particular affinity for N‐acetylgalactosamine residues on GPI‐anchored proteins (Pardo‐Lopez *et al*., 2006; Ning *et al*., 2010). This study identified two membrane alkaline phosphatases associated with the *T. ni* BBMV; both have a GPI anchor motif and are potential receptors for *B. thuringiensis* delta‐endotoxins.

Two isoforms of FE4 carboxylesterase were associated with the BBMV. In addition to their role in digestion, these enzymes are capable of cleaving ester bonds in other organic molecules, including certain classes of insecticides. Indeed, amplification of genes encoding insecticide detoxifying enzymes, namely glutathione‐S‐transferase, cytochrome P450 monooxygenases and esterases, is a major cause of insecticide resistance (Bass & Field, 2011). In *Myzus persicae*, gene amplification leading to increased expression of FE4 esterase and the closely related E4 esterase confers resistance of the insect to organophosphate insecticides (Field & Devonshire, 1998). While insecticide resistance arising from increased esterase activity has been commonly reported in hemipterans and dipterans, the presence of these enzymes in *T. ni* suggests that this mechanism may also available to lepidopteran pests.

### Transporters

#### Nutrient transporters

The BBM is the principal site for nutrient acquisition and many of the proteins that were found to be associated with the *T. ni* BBMVs are likely involved in some aspect of nutrient molecule transport. According to the protein family database (Pfam; www.sangar.ac.uk/Software/Pfam/), the *Drosophila melanogaster* genome encodes 44 proteins with sugar transporter motifs. This study found 15 proteins associated with the *T. ni* BBMVs with similarity to the facilitated trehalose transporter 1 (TreT1). TreT1 transporters are highly specific for trehalose and do not recognize other disaccharides. Moreover, these transporters facilitate trehalose transport independent of membrane potential (Kikawada *et al*., 2007). Trehalose is the major hemolymph disaccharide sugar in most insects. It serves as a readily accessible energy source, such as during flight (Shulka *et al*., 2015), but is also used as a desiccation and cryoprotectant (Kikawada *et al*., 2007). Trehalose is synthesized in the insect fat body; however, it may also be present in the diet as plants use trehalose as an osmoprotectant in response to salt and drought stress (Grennan, 2007). The association of trehalose transporters with the *T. ni* midgut epithelial membrane indicate that both *de novo* synthesis and acquisition from external sources may contribute to the trehalose pool within the hemolymph. The hydrolysis of trehalose by trehalase to produce to two glucose molecules is an early and important regulatory step in the chitin biosynthetic pathway (Shen *et al*., 2017). While the midgut is not covered in a cuticle, it does secrete chitin, which forms a scaffold that supports the peritrophic matrix (Hegedus *et al*., 2009). Three membrane‐associated enzymes were identified in the BBMV fraction that are involved in some aspect of trehalose, chitin or cuticle metabolism; trehalase‐like isoform x1 (chitin synthesis), chitinase‐3‐like protein 2 (hydrolysis), and laccase‐1‐like isoform x2 (sclerotization).

Transporters for other carbohydrates, such as glucose, were also associated with the BBMV, as were five monocarboxylate transporters. The latter transporters are members of a family of multiple pass transmembrane proteins, which carry molecules with a single carboxylate group, for example pyruvate and lactate, across the plasma membrane in a proton‐dependent manner (Halestrap, 2012). Several amino acid transporters, as well as two long chain fatty acid transporters and a nucleoside transporter were also identified. Membrane transporters were also present to facilitate the acquisition of folate and thiamine from the diet, and a sodium‐dependent multi‐vitamin transporter may be capable of transporting a variety of vitamins and other cofactors, such as biotin, pantothenic acid and lipoic acid (Vadlapudi *et al*., 2012).

#### Ion transporters

The cell membrane is not permeable to most ions so their movement into or out of the cell requires specific transporter proteins. Seven proteins similar to organic cation transporters were associated with the *T. ni* BBMVs. Organic cationic transporters translocate cations, such as monoamines, coenzymes, and other molecules across the cell membrane (Koepsell *et al*., 2003). Many transporters capable of transporting inorganic cations were also identified. These included six zinc transporters, several potassium and/or calcium transporters and a bevy of copper and other metal transporters. The importance of phosphate is exemplified by the presence of seven inorganic phosphate transporters. Transporters for other anions were also present, including three band 3 anion transport proteins (solute carrier family 4, member 1), which exchange chloride with bicarbonate across plasma membranes. A bestrophin 4 protein was also identified that allows for calcium‐dependent transport of chloride ions (Milenkovic *et al*., 2008).

Four proteins were identified as being similar to the vacuolar (V‐type) proton ATPase. V‐type proton ATPases have previously been reported in midgut tissue from *Heliothis virescens* (Krishnamoorthy *et al*., 2007), *H. armigera* (Yuan *et al*., 2011), *Bombyx mori* (Kajiwara *et al*., 2005), and *Manduca sexta* (Pauchet *et al*., 2009). V‐type proton ATPases are localized on the apical surface of midgut cells and use energy from ATP hydrolysis to produce a proton gradient across the insect midgut epithelium, which facilitates the activity of other molecule transporters (Wieczorek *et al*., 1999). It is also possible that proton ATPases help to regulate gut pH as the midgut of *T. ni* larvae, like other lepidopterans, is highly basic (Braun & Keddie, 1997) and the digestive enzyme complement of lepidopteran larvae is most active at elevated pH (Hegedus *et al*., 2003). V‐type proton ATPase subunits in *Heliothis virescens* midgut were shown to interact with the *B. thuringiensis* toxin Cry1Ac (Krishnamoorthy *et al*., 2007); however, *P. interpunctella* larvae resistant to Cry1Ab toxin had higher levels of V‐ATPase subunit B in the midgut epithelium (Candas *et al*., 2003).

#### ATP‐binding cassette (ABC) and major facilitator superfamily (MFS) transporters

ABC and MFS transporters exhibit a wide range of specificities including polysaccharides, drugs, sugars, heavy metals, peptides, amino acids, and inorganic ions (Perlin *et al*., 2014). ABC transporters are the largest and most widely expressed family of transporters and hydrolyze ATP to energize the transport of molecules across the cell membrane (Leslie *et al*., 2005). Eighteen proteins associated with the *T. ni* BBMVs were members of the ABC family of transporters. Thirteen of the ABC transporters were similar to multidrug resistance associated proteins (MDRP), which have been implicated in the efflux of host plant phytoalexins in phytopathogens (Perlin *et al*., 2014). The number of such transporters in the *T. ni* midgut epithelium may be reflective of its cosmopolitan, oligophagous nature compared to insects with more restricted host specificities.

#### Other transporters

Five proteins identified in the BBMVs had similarity to, nose‐resistant to fluoxetine protein‐6 (NRF‐6). The NRF‐6 protein is a multipass membrane protein and has been best studied in the invertebrate model organism *Caenorhabditis elegans* where, among other functions, it is involved in the uptake of a range of molecules, including lipids and xenobiotic compounds from the intestine (Choy *et al*., 2006; Watts & Browse, 2006).

Three SID‐1‐like double stranded RNA (dsRNA) transporters were associated with the BBMVs. SID‐1 is required for the passive uptake of dsRNA from surrounding medium and for the movement of RNA interference signals between cells (Shih & Hunter, 2011). This is interesting since experiments to induce systemic RNAi in lepidopteran insects has met with limited success (Terenius *et al*., 2010), while feeding of dsRNA to larvae results in effective silencing of midgut genes (Toprak *et al*., 2013). It remains to be determined what might be the significance of dsRNA uptake by the midgut epithelium in insect biology and/or insect–host plant interactions.

This study identified two aquaporins that were associated with the BBMV. Aquaporins are membrane spanning proteins that form water channels through the cell membrane. They are an integral component of the system that maintains water homeostasis which is critical for the establishment of concentration gradients and osmotic balance. Aquaporins have also been implicated in freezing and desiccation tolerance in some insects (Cohen, 2012).

### Proteins involved in cell signaling and development

The BBMV‐enriched fraction will contain some remnants of the columnar cell membrane, and several proteins involved in cell signaling, development or cell–cell association were identified and are described below. The midgut epithelium is constantly being sloughed and renewed. In addition, the cellular composition (columnar cells, goblet cells, and underlying regenerative cells) of the *T. ni* midgut changes along its length (Engelhard *et al*., 1991; Braun, 1996). As such, some of the proteins associated with the BBMV fraction were involved in aspects of signaling or cell–cell interaction related to cellular development and morphogenesis. A G‐protein coupled receptor (GPCR) 125 was identified that was highly similar to the human adhesion GPCR which stimulates tumor angiogenesis and is involved in other aspects of metazoan development (Weis & Cheresh, 2011). A BBMV GPCR similar to Methuselah (mth2) was identified which may regulate cell longevity in response to stress (InterPro: IPR010596); this would be an important consideration for midgut epithelial cell turnover. Notch, a cell surface transmembrane protein that regulates cellular events, such as differentiation, proliferation, and apoptosis (Guruharsha *et al*., 2012), was found in the BBMV enriched fraction. The Hedgehog signaling pathway is also important for determining cell fate and morphogenesis (Ingham *et al*., 2011). Hedgehog secretion is dependent upon the membrane protein Dispatched (Tukachinsky *et al*., 2012), which was found in the BBMV fraction, as was the Hedgehog receptor Patched. Independent of the Notch and Hedgehog signaling pathways, Fascilin 2, the neural cell‐adhesion molecule, specifically inhibits epidermal growth factor‐mediated signaling during development (Mao & Freeman, 2009). Three Fascilin 2 proteins were found in the BBMVs. In *Drosophila*, the semaphorin receptor Plexin A is involved in axon development and other aspects related to development (Xu *et al*., 2000). Plexin A and the Plexin A domain‐containing protein 2‐like, which has also been implicated in cell morphogenesis, were associated with the BBMVs. A rolling stone‐like transmembrane protein was also identified, which in *Drosophila*, is involved in myogenesis (Paululat *et al*., 1997).

### Proteins involved in cell–cell interactions

Two integrin beta‐nu proteins and two integrin alpha isoforms were found in the BBMV fraction. Integrins are transmembrane proteins that connect the extracellular matrix to the cytoskeleton. In insects, integrin beta‐nu is required for migration of primordial midgut cells and for maintaining cell polarity in the midgut epithelium. It is also involved in maintaining endodermal integrity and adhesion of the midgut epithelium to the surrounding muscle (Devenport & Brown, 2004). A dystroglycan was also identified. Dystroglycan is the transmembrane component of the dystroglycan complex that, like integrins, links the extracellular matrix to the actin cytoskeleton (Adams & Brancaccio, 2015). Two cadherins were found in the BBMV fraction. Cadherins are calcium‐dependent cell adhesion transmembrane proteins. The extracellular domains interact to form “adherens” junctions which bind cells within tissues together, while the intracellular domain interacts with other partners to anchor the adherens to actin (Harris & Tepass, 2010). Binding of *B. thuringiensis* delta‐endotoxin to cadherin is thought to facilitate interaction with GPI‐anchored membrane proteins, such as APN and alkaline phosphatase (Pardo‐Lopez *et al*., 2006). A lachesin‐like isoform x1 protein was found in the BBMV fraction. Lachesins are another type of cell adhesion protein and in *Drosophila* regulate cell size and cell adhesion during morphogenesis (Llimargas *et al*., 2004). An innexin was found in the BBMV fraction. Tight association of cells is necessary not only for maintaining tissue integrity, but also to allow for cell‐to‐cell communication. In this regard, innexins form gap junctions between cells that permit the exchange of ions and small molecules (Bao *et al*., 2007).

### Other BBMV proteins

Other proteins associated with the BBMV plasma membrane may be involved in some aspect of defense against enteric pathogens, these included two peptidoglycan‐recognition proteins, which are expressed in response to bacterial infections and induce the production of antimicrobial peptides, promote phagocytosis or act directly to hydrolyze peptidoglycan (Dziarski & Gupta, 2006). An isoform of Croquemort was associated with the BBMVs. Croquemort is a type of CD36 receptor which in macrophages is required for phagocytosis of apoptotic cells. Although it is not required for engulfment of bacteria, members of this family are part of the ancient innate immune system common to vertebrates and invertebrates (France *et al*., 1999). Two proteins similar to transmembrane protein 120 were associated with the BBMVs, which are described as also being involved in the innate immune response against Gram‐negative bacteria (www.uniprot.org/uniprot/Q9U1M2).

## Discussion

The midgut epithelium's primary role is for the uptake and acquisition of nutrients from the lumen. The analysis of the *T. ni* BBM presented here identified several membrane‐associated enzymes involved in the terminal stages of macromolecule digestion, as well as a plethora of transporters needed to translocate small molecules across the epithelial cell membrane. The midgut epithelium is also the principal site for interaction with host‐derived anti‐nutritional molecules and toxins. Indeed, transporters were identified that have been implicated in the efflux and metabolism of host plant phytoalexins and other xenobiotics. Importantly, the midgut epithelium is a target for pathogens and several membrane proteins corresponding to known receptors for *B. thuringiensis* toxin (aminopeptidase‐N, cadherin, V‐type proton ATPase) were found. Finally, the insect virus *per os* infectivity (pif) complex is believed to interact with a ligand present on the epithelial cell membrane; however, this has yet to be identified for any baculovirus system. Should this model hold true, it is likely that the ligand(s) recognized by the pif complex is one of the membrane proteins described in this study. This is the most detailed proteomic analysis of the lepidopteran midgut epithelial cell membrane to date and will provide information and opportunities to better understand the biochemical, physiological, and pathological processes occurring in the larval midgut.

## Supporting information


**Supporting Information 1** BBMV protein sequences.

**Supporting Information 2** BBMV protein nucleotide sequences.

**Supporting Information 3** BBMV membrane protein sequences.

**Supporting Information 4** BBMV membrane protein nucleotide sequences.

**Table S1** *T. ni* BBMV proteins.

**Table S2** Putative *T. ni* BBMV and epithelial membrane proteins.

## References

[ins12547-bib-0001] Abdul‐Rauf, M. and Ellar, D.J. (1999) Isolation and characterization of brush border membrane vesicles from whole *Aedes aegypti* larvae. Journal of Invertebrate Pathology, 73, 45–51. 9878289 10.1006/jipa.1998.4792

[ins12547-bib-0002] Adams, J.C. and Brancaccio, A. (2015) The evolution of the dystroglycan complex, a major mediator of muscle integrity. Biology Open, 4, 1163–1179. 26319583 10.1242/bio.012468 PMC4582122

[ins12547-bib-0003] Bao, L. , Samuels, S. , Locovei, S. , MacAgno, E. , Muller, K. and Dahl, G. (2007) Innexins form two types of channels. FEBS Letters, 581, 5703–5708. 18035059 10.1016/j.febslet.2007.11.030 PMC2489203

[ins12547-bib-0004] Bass, C. and Field, L.M. (2011) Gene amplification and insecticide resistance. Pest Management Science, 67, 886–890. 21538802 10.1002/ps.2189

[ins12547-bib-0005] Bolognesi, R. , Terra, W. and Ferreira, C. (2008) Peritrophic membrane role in enhancing digestive efficiency. Journal of Insect Physiology, 54, 1413–1422. 18761346 10.1016/j.jinsphys.2008.08.002

[ins12547-bib-0075] Braun, L. (1996) A new tissue model for evaluating effects of *Bacillus thuringiensis* toxins on insect midgut epithelium. Ph.D. Thesis, University of Alberta, Canada. 10.1006/jipa.1996.4632 9056459

[ins12547-bib-0006] Braun, L. and Keddie, B.A. (1997) A new tissue technique for evaluating effects of *Bacillus thuringiensis* toxins on insect midgut epithelium. Journal of Invertebrate Pathology, 69, 92–104. 9056459 10.1006/jipa.1996.4632

[ins12547-bib-0007] Campbell, P.M. , Cao, A.T. , Hines, E.R. , East, P.D. and Gordon, K.H.J. (2008) Proteomic analysis of the peritrophic matrix from the gut of the caterpillar, *Helicoverpa armigera* . Insect Biochemistry and Molecular Biology, 38, 950–958. 18760362 10.1016/j.ibmb.2008.07.009

[ins12547-bib-0008] Candas, M. , Loseva, O. , Oppert, B. , Kosaraju, P. and Bulla, L.A. (2003) Insect resistance to *Bacillus thuringiensis* . Molecular Cellular Proteomics, 2, 19–28. 12601079 10.1074/mcp.m200069-mcp200

[ins12547-bib-0009] Chen, Y.R. , Zhong, S.L. , Fei, Z.J. , Gao, S. , Zhang, S.Y. , Li, Z.F. *et al* (2014) Transcriptome responses of the host *Trichoplusia ni* to infection by the baculovirus *Autographa californica* multiple nucleopolyhedrovirus. Journal of Virology, 88, 13781–13797. 25231311 10.1128/JVI.02243-14 PMC4248991

[ins12547-bib-0010] Choy, R.K.M. , Kemner, J.M. , Thomas, J.H. (2006) Fluoxetine‐resistance genes in *Caenorhabditis elegans* function in the intestine and may act in drug transport. Genetics, 172, 885–892. 16118202 10.1534/genetics.103.024869 PMC1456238

[ins12547-bib-0011] Cohen, E. (2012) Roles of aquaporins in osmoregulation, desiccation and cold hardiness in insects. Entomology, Ornithology and Herpertology, S1, 001

[ins12547-bib-0012] De Veau, E.J.I. and Schultz, J.C. (1992) Reassessment of interaction between gut detergents and tannins in Lepidoptera and significance for gypsy moth larvae. Journal of Chemical Ecology, 18, 1437–1453. 24254217 10.1007/BF00994367

[ins12547-bib-0013] Devenport, D. and Brown, N.H. (2004) Morphogenesis in the absence of integrins: mutation of both *Drosophila* beta subunits prevents midgut migration. Development, 131, 5405–5415. 15469969 10.1242/dev.01427

[ins12547-bib-0014] Dziarski, R. and Gupta, D. (2006) The peptidolycan recognition proteins (PGRPs). Genome Biology, 7, e232. 10.1186/gb-2006-7-8-232 PMC1779587 16930467

[ins12547-bib-0015] Engelhard, E.K. , Keddie, B.A. and Volkman, L.E. (1991) Isolation of third, fourth, and fifth instar larval midgut epithelia of the moth, *Trichoplusia ni* . Tissue and Cell, 23, 917–928. 18621194 10.1016/0040-8166(91)90041-q

[ins12547-bib-0016] Field, L.M. and Devonshire, A.L. (1998) Evidence that the E4 and FE4 esterase genes responsible for insecticide resistance in the aphid *Myzus persicae* (Sulzer) are part of a gene family. Biochemical Journal, 330, 169–173. 9461506 10.1042/bj3300169 PMC1219123

[ins12547-bib-0017] Finn, R.D. , Clements, J. , Arndt, W. , Miller, B.L. , Wheeler, T.J. , Schreiber, F. *et al* (2015) HMMER web server: 2015 update. Nucleic Acids Research Web Server, 43, W30–W38. 10.1093/nar/gkv397 PMC4489315 25943547

[ins12547-bib-0018] Franc, N.C. , Heitzler, P. , Ezekowitz, R.A. and White, K. (1999) Requirement for croquemort in phagocytosis of apoptotic cells in *Drosophila* . Science, 284, 1991–1994. 10373118 10.1126/science.284.5422.1991

[ins12547-bib-0019] Harris, T.J. and Tepass, U. (2010) Adherens junctions: from molecules to morphogenesis. Nature Reviews in Molecular and Cell Biology, 11, 502–514. 20571587 10.1038/nrm2927

[ins12547-bib-0020] Gai, Z.C. , Zhang, X.J. , Wang, X. , Peng, J.X. , Li, Y. , Liu, K.Y. *et al* (2013) Differential proteomic analysis of *Trichoplusia ni* cells after continuous selection with activated Cry1Ac toxin. Cytotechnology, 65, 425–435. 23070538 10.1007/s10616-012-9496-4 PMC3597167

[ins12547-bib-0021] Garczynski, S.F. and Adang, M.J. (1995) *Bacillus thuringiensis* Cry1Ac delta‐endotoxin binding aminopeptidase in the *Manduca sexta* midgut has a glycosylphosphatidylinositol anchor. Insect Biochemistry and Molecular Biology, 25, 409–415

[ins12547-bib-0022] Granados, R.R. and Lawler, K.A. (1981) *In vivo* pathway of *Autographa californica* baculovirus invasion and infection. Virology, 108, 297–308. 18635031 10.1016/0042-6822(81)90438-4

[ins12547-bib-0023] Grennan, A.K. (2007) The role of trehalose biosynthesis in plants. Plant Physiology, 144, 3–5. 17494918 10.1104/pp.104.900223 PMC1913774

[ins12547-bib-0024] Guruharsha, K.G. , Kankel, M.W. and Artavanis‐Tsakonas, S. (2012) The Notch signalling system: recent insights into the complexity of a conserved pathway. Nature Reviews in Genetics, 13, 654–666. 10.1038/nrg3272 PMC4369923 22868267

[ins12547-bib-0025] Halestrap, A.P. (2012) The monocarboxylate transporter family—structure and functional characterization. IUBMB Life, 64, 1–9. 22131303 10.1002/iub.573

[ins12547-bib-0026] Haas‐Stapleton, E.J. , Washburn, J.O. and Volkman, L.E. (2004) P74 mediates specific binding of *Autographa californica* M nucleopolyhedrovirus occlusion‐derived virus to primary cellular targets in the midgut epithelia of *Heliothis virescens* larvae. Journal of Virology, 78, 6786–6791. 15194753 10.1128/JVI.78.13.6786-6791.2004 PMC421674

[ins12547-bib-0027] Hegedus, D.D. , Baldwin, D. , O'Grady, M. , Braun, L. , Gleddie, S. , Sharpe, A. *et al* (2003) Midgut proteases from *Mamestra configurata* (Lepidoptera: Noctuidae) larvae: characterization, cDNA cloning and expressed sequence tag analysis. Archives of Insect Biocheistry and Physiology, 53, 30–47. 10.1002/arch.10084 12701112

[ins12547-bib-0028] Hegedus, D.D. , Erlandson, M. , Gillott, C. and Toprak, U. (2009) New insights into peritrophic matrix synthesis, architecture and function. Annual Reviews of Entomology, 54, 285–302. 10.1146/annurev.ento.54.110807.090559 19067633

[ins12547-bib-0029] Horton, H.M. and Burand, J.P. (1993) Saturable attachment sites for polyhedron‐derived baculovirus on insect cells and evidence for entry via direct membrane fusion. Journal of Virology, 67, 1860–1868. 8445715 10.1128/jvi.67.4.1860-1868.1993 PMC240252

[ins12547-bib-0030] Ingham, P.W. , Nakano, Y. and Seger, C. (2011) Mechanisms and functions of Hedgehog signalling across the metazoa. Nature Reviews in Genetics, 12, 393–406. 10.1038/nrg2984 21502959

[ins12547-bib-0076] Javed, M.A. , Harris, S. , Willis, L. , Theilmann, D. , Donly, C. , Hegedus, D.D. and Erlandson, M. (2016) Microscopic investigation of AcMNPV infection in the *Trichoplusia ni* midgut. Journal of Invertebrate Pathology, 141, 24–33. 27793742 10.1016/j.jip.2016.10.006

[ins12547-bib-0031] Kajiwara, H. , Ito, Y. , Imamaki, A. , Nakamura, M. , Mita, K. and Ishizaka, M. (2005) Protein profile of silkworm midgut of fifth‐instar day‐3 larvae. Journal of Electrophoresis, 49, 61–69.

[ins12547-bib-0032] Keddie, B.A. , Aponte, G.W. and Volkman, L.E. (1989) The pathway of infection of *Autographa californica* nuclear polyhedrosis virus in an insect host. Science, 243, 1728–1730. 2648574 10.1126/science.2648574

[ins12547-bib-0033] Kikawada, T. , Saito, A. , Kanamori, Y. , Nakahara, Y. , Iwata, K. , Tanaka, D. *et al* (2007) Trehalose transporter 1, a facilitated and high‐capacity trehalose transporter, allows exogenous trehalose uptake into cells. Proceedings of the National Academy of Sciences USA, 104, 11585–11590. 10.1073/pnas.0702538104 PMC1905927 17606922

[ins12547-bib-0034] Knebel‐Morsdorf, D. , Flipsen, J.T. , Roncarati, R. , Jahnel, F. , Kleefsman, A.W. and Vlak, J.M. (1996) Baculovirus infection of *Spodoptera exigua* larvae: *lacZ* expression driven by promoters of early genes *pe38* and *me53* in larval tissue. Journal of General Virology, 77, 815–824. 8609477 10.1099/0022-1317-77-5-815

[ins12547-bib-0035] Knight, P.J. , Knowles, B.H. and Ellar, D.J. (1994) The receptor for *Bacillus thuringiensis* CrylA(c) delta‐endotoxin in the brush border membrane of the lepidopteran *Manduca sexta* is aminopeptidase N. Molecular Microbiology, 11, 429–436. 7908713 10.1111/j.1365-2958.1994.tb00324.x PMC7168503

[ins12547-bib-0036] Koepsell, H. , Schmitt, B.M. and Gorboulev, V. (2003) Organic cation transporters. Reviews of Physiology, Biochemistry and Pharmacology, 150, 36–90. 10.1007/s10254-003-0017-x 12827517

[ins12547-bib-0037] Krishnamoorthy, M. , Jurat‐Fuentes, J.L. , McNall, R.J. , Andacht, T. and Adang, M.J. (2007) Identification of novel Cry1Ac binding proteins in midgut membranes from *Heliothis virescens* using proteomic analyses. Insect Biochemistry and Molecular Biology, 37, 189–201. 17296494 10.1016/j.ibmb.2006.10.004

[ins12547-bib-0038] Lagarda‐Diaz, I. , Guzman‐Partida, A.M. , Huerta‐Ocampo, J.A. , Winzerling, J. and Vazquez‐Moreno, L. (2016) Identification of membrane proteins of the midgut of *Zabrotes subfasciatus* larvae associated with the insecticidal mechanism of PF2 lectin. Journal of Asia‐Pacific Entomology, 19, 677–682.

[ins12547-bib-0039] Leslie, E.M. , Deeley, R.G. and Cole, S.P. (2005) Multidrug resistance proteins: role of P‐glycoprotein, MRP1, MRP2, and BCRP (ABCG2) in tissue defense. Toxicology and Applied Pharmacology, 204, 216–237. 15845415 10.1016/j.taap.2004.10.012

[ins12547-bib-0040] Lehane, M.J. and Billingsley, P.F. (1996) Biology of the Insect Midgut. Chapman and Hall, London.

[ins12547-bib-0041] Linz, L.B. , Liu, S. , Chougule, N.P. and Bonning, B.C. (2015) *In vitro* evidence supports membrane alanyl aminopeptidase N as a receptor for a plant virus in the pea aphid vector. Journal of Virology, 89, 11203–11212. 26311872 10.1128/JVI.01479-15 PMC4645670

[ins12547-bib-0042] Llimargas, M. , Strigini, M. , Katidou, M. , Karagogeos, D. and Casanova, J. (2004) Lachesin is a component of a septate junction‐based mechanism that controls tube size and epithelial integrity in the *Drosophila* tracheal system. Development, 131, 181–190. 14681183 10.1242/dev.00917

[ins12547-bib-0043] Ma, W.H. , Zhang, Z. , Peng, C.H. , Wang, X.P. , Li, F. and Lin, Y.J. (2012) Exploring the midgut transcriptome and brush border membrane vesicle proteome of the rice stem borer, *Chilo suppressalis* (Walker). PLoS ONE, 7, e38151. 22666467 10.1371/journal.pone.0038151 PMC3362559

[ins12547-bib-0044] Mao, Y. and Freeman, M. (2009) Fascilin 2, the *Drosophila* orthologue of neural cell‐adhesion molecule, inhibits EGF receptor signalling. Development, 136, 473–481. 19141676 10.1242/dev.026054 PMC2687591

[ins12547-bib-0045] McNall, R.J. and Adang, M.J. (2003) Identification of novel *Bacillus thuringiensis* Cry1Ac binding proteins in *Manduca sexta* midgut through proteomic analysis. Insect Biochemistry and Molecular Biology, 33, 999–1010. 14505693 10.1016/s0965-1748(03)00114-0

[ins12547-bib-0046] Milenkovic, V.M. , Langmann, T. , Schreiber, R. , Kunzelmann, K. and Weber, B.H. (2008) Molecular evolution and functional divergence of the bestrophin protein family. BMC Evolutionary Biology, 8, e72. 10.1186/1471-2148-8-72 PMC2292144 18307799

[ins12547-bib-0077] Ning, C. , Wu, K. , Liu, C. , Gao, Y. , Jurat‐Fuentes, J.L. and Go, X. (2010) Characterization of a Cry1Ac toxin‐binding alkaline phosphatase in the midgut from Helicoverpa armigera (Hübner) larvae. Journal of Insect Physiology, 56, 666–672. 20170658 10.1016/j.jinsphys.2010.02.003

[ins12547-bib-0047] Pauchet, Y. , Muck, A. , Svatoš, A. and Heckel, D.G. (2009) Chromatographic and electrophoretic resolution of proteins and protein complexes from the larval midgut microvilli of *Manduca sexta* . Insect Biochemistry and Molecular Biology, 39, 467–474. 19464367 10.1016/j.ibmb.2009.05.001

[ins12547-bib-0048] Paululat, A. , Goubeaud, A. , Damm, C. , Knirr, S. , Burchard, S. and Renkawitz‐Pohl, R. (1997) The mesodermal expression of rolling stone (rost) is essential for myoblast fusion in *Drosophila* and encodes a potential transmembrane protein. Journal of Cell Biology, 138, 337–348. 9230076 10.1083/jcb.138.2.337 PMC2138187

[ins12547-bib-0049] Parker, C.E. , Warren, M.R. , Loiselle, D.R. , Dicheva, N.N. , Scarlett, C.O. and Borchers, C.H. (2005) Identification of components of protein complexes. Methods in Molecular Biology, 301, 117–151. 15917630 10.1385/1-59259-895-1:117

[ins12547-bib-0050] Pardo‐Lopez, L. , Gomez, I. , Rausell, C. , Sanchez, J. , Soberon, M. and Bravo, A. (2006) Structural changes of the Cry1Ac oligomeric pre‐pore from *Bacillus thuringiensis* induced by N‐acetylgalactosamine facilitates toxin membrane insertion. Biochemistry, 45, 10329–10336. 16922508 10.1021/bi060297z

[ins12547-bib-0051] Peng, K. , van Lent, J.W.M. , Boeren, S. , Fang, M. , Theilmann, D.A. , Erlandson, M.A. *et al* (2013) Characterization of novel components of the baculovirus *per os* infectivity factor complex. Journal of Virology, 86, 4981–4988. 10.1128/JVI.06801-11 PMC3347349 22379094

[ins12547-bib-0052] Perlin, M.H. , Andrews, J. and Toh, S.S. (2014) Essential letters in the fungal alphabet: ABC and MFS transporters and their roles in survival and pathogenicity. Advances in Genetics, 85, 201–253. 24880736 10.1016/B978-0-12-800271-1.00004-4

[ins12547-bib-0053] Popova‐Butler, A. and Dean, D.H. (2009) Proteomic analysis of the mosquito *Aedes aegypti* midgut brush border membrane vesicles. Journal of Insect Physiology, 55, 264–272. 19133270 10.1016/j.jinsphys.2008.12.008 PMC2735124

[ins12547-bib-0054] Sangadala, S. , Walters, F.S. , English, L.H. and Adang, M.J. (1994) A mixture of *Manduca sexta* aminopeptidase and phosphatase enhances *Bacillus thuringiensis* insecticidal CryIA(c) toxin binding and 86Rb(+)‐K+ efflux *in vitro* . Journal of Biological Chemistry, 269, 10088–10092. 8144508

[ins12547-bib-0055] Shen, Q.D. , Yang, M.M. , Xie, G.Q. , Wang, H.J. , Zhang, L. , Qiu, L.Y. *et al* (2017) Excess trehalose and glucose affects chitin metabolism in brown planthopper (*Nilaparvata lugens*). Journal of Asia‐Pacific Entomology, 20, 449–455.

[ins12547-bib-0056] Shih, J.D. and Hunter, C.P. (2011) SID‐1 is a dsRNA‐selective dsRNA‐gated channel. RNA, 17, 1057–1065. 21474576 10.1261/rna.2596511 PMC3096038

[ins12547-bib-0057] Shulka, E. , Thorat, L.J. , Nath, B.B. and Gaikwad, S.M. (2015) Insect trehalase: physiological significance and potential applications. Glycobiology, 25, 357–367. 25429048 10.1093/glycob/cwu125

[ins12547-bib-0058] Simpson, R.M. , Newcomb, R.D. , Gatehouse, H.S. , Crowhurst, R.N. , Chagné, D. , Gatehouse, L.N. *et al* (2007) Expressed sequence tags from the midgut of *Epiphyas postvittana* (Walker) (Lepidoptera: Tortricidae). Insect Molecular Biology, 16, 675–690. 18092997 10.1111/j.1365-2583.2007.00763.x

[ins12547-bib-0059] Sutherland, D.W.S. and Greene, G.L. (1984) Cultivated and wild host plants Suppression and Management of Cabbage Looper Populations (eds. Lingren P.D. & Greene G.L.), pp. 1–13. U.S. Department of Agriculture, Technical Bulletin no. 1684, U.S.A.

[ins12547-bib-0060] Terenius, O. , Papanicolaou, A. , Garbutt, J.S. , Eleftherianos, I. , Huvenne, H. , Kanginakudru, S. *et al* (2010) RNA interference in Lepidoptera—successes and failures. Insect Biochemistry and Molecular Biology, 57, 231–245.

[ins12547-bib-0061] Terra, W.R , Ferreira, C. , Jordao, B.P. and Dillon, R.J. (1994) Digestive enzymes Biology of the Insect Midgut (eds. Lehane M.J. & Billingsely P.F.). Chapman and Hall, London.

[ins12547-bib-0062] Toprak, U. , Erlandson, M. , Baldwin, D. , Karcz, S. , Wan, L. , Coutu, C. *et al* (2015) Identification of the *Mamestra configurata* (Lepidoptera: Noctuidae) peritrophic matrix proteins and enzymes involved in peritrophic matrix chitin metabolism. Insect Science, 23, 656–674. 25846407 10.1111/1744-7917.12225

[ins12547-bib-0063] Toprak, U. , Baldwin, D. , Erlandson, M. , Gillott, C. , Harris, S. and Hegedus, D.D. (2013) *In vitro* and *in vivo* application of RNA interference for targeting genes involved in peritrophic matrix synthesis in a lepidopteran system. Insect Science, 20, 92–100. 23955829 10.1111/j.1744-7917.2012.01562.x

[ins12547-bib-0064] Tukachinsky, H. , Kuzmickas, R.P. , Jao, C.Y. , Liu, J. and Salic, A. (2012) Dispatched and Scube mediate the efficient secretion of the cholesterol‐modified Hedgehog ligand. Cell, 30, 308–320. 10.1016/j.celrep.2012.07.010 PMC3682496 22902404

[ins12547-bib-0065] Vadlapudi, A.D. , Vadlapatla, R.K. and Mitra, A.K. (2012) Sodium dependent multivitamin transporter (SMVT): a potential target for drug delivery. Current Drug Targets, 13, 994–1003. 22420308 10.2174/138945012800675650 PMC4406285

[ins12547-bib-0066] Vail, P.V. , Anderson, S.J. and Jay, D.L. (1973) New procedures for rearing cabbage loopers and other lepidopterous larvae for progagation of nuclear polyhedrosis viruses. Environmental Entomology, 2, 339–334.

[ins12547-bib-0067] Wang, P. , Zhang, X. and Zhang, J. (2005) Molecular characterization of four midgut aminopeptidase N isozymes from the cabbage looper, Trichoplusia ni. Insect Biochemistry and Molecular Biology, 35, 611–620. 15857766 10.1016/j.ibmb.2005.02.002

[ins12547-bib-0068] Watts, J.L. and Browse, J. (2006) Dietary manipulation implicates lipid signaling in the regulation of germ cell maintenance in *C. elegans* . Developmental Biology, 292, 381–392. 16487504 10.1016/j.ydbio.2006.01.013 PMC1584401

[ins12547-bib-0069] Weis, S.M. and Cheresh, D.A. (2011) Tumor angiogenesis: molecular pathways and therapeutic targets. Nature Medicine, 17, 1359–1370. 10.1038/nm.2537 22064426

[ins12547-bib-0070] Wieczorek, H. , Grube, G. , Harvey, W.R. , Huss, M. and Merzendorfer, H. (1999) The plasma membrane H+‐V‐ATPase from tobacco hornworm midgut. Journal of Bioenergetics and Biomembranes, 31, 67–74. 10340850 10.1023/a:1005448614450

[ins12547-bib-0071] Wolfersberger, M. , Luethy, P. , Maurer, A. , Parenti, P. , Sacchi, F.V. , Giordana, B. *et al* (1987) Preparation and partial characterization of amino acid transporting brush border membrane vesicles from the larval midgut of the cabbage butterfly (*Pieris brassicae*). Comparative Biochemistry and Physiology, 86, 301–308.

[ins12547-bib-0072] Xu, X.M. , Fisher, D.A. , Zhou, L. , White, F.A. , Ng, S. , Snider, W.D. *et al* (2000) The transmembrane protein Semaphorin 6A repels embryonic sympathetic axons. Journal of Neuroscience, 20, 2638–2648. 10729344 10.1523/JNEUROSCI.20-07-02638.2000 PMC6772238

[ins12547-bib-0073] Yachdav, G. , Kloppmann, E. , Kajan, L. , Hecht, M. , Goldberg, T. , Hamp, T. *et al* (2014) PredictProtein: an open resource for online prediction of protein structural and functional features. Nucleic Acids Research, 42, W337–W343. 24799431 10.1093/nar/gku366 PMC4086098

[ins12547-bib-0074] Yuan, C. , Ding, X.Z. , Xia, L.Q. , Yin, J. , Huang, S.Y. and Huang, F. (2011) Proteomic analysis of BBMV in *Helicoverpa armigera* midgut with and without Cry1Ac toxin treatment. Biocontrol Science and Technology, 21, 139–151.

